# Women’s advice to healthcare professionals regarding breastfeeding: “offer sensitive individualized breastfeeding support”- an interview study

**DOI:** 10.1186/s13006-019-0247-4

**Published:** 2019-12-16

**Authors:** Ingrid Blixt, Margareta Johansson, Ingegerd Hildingsson, Zoi Papoutsi, Christine Rubertsson

**Affiliations:** 1 Department of Women’s and Children’s Health, Akademiska sjukhuset, Uppsala University, 751 85 Uppsala, Sweden; 2 Centre for Clinical Research Sörmland, Uppsala University, Sveavägen entré 9 631 88, Eskilstuna, Sweden; 3 Department of Health Science Faculty of Medicine, Lund University, Lund, Sweden

**Keywords:** Breastfeeding, Healthcare professionals, Support, Women’s advice

## Abstract

**Background:**

The World Health Organization recommends exclusive breastfeeding for 6 months followed by continued breastfeeding with complementary food up to 2 years of age or beyond. Few women achieve this recommendation in Sweden, and they often stop breastfeeding earlier than they would like. Investigating women’s advice to healthcare professionals is important for the provision of optimal breastfeeding support. The aim of this study was to explore women’s advice to healthcare professionals regarding support for continuing to breastfeed for at least 6 months.

**Methods:**

This investigation used an exploratory study design, and a purposive sample of women was recruited between 2015 and 2016 through social media platforms. The work is a follow-up of an earlier study exploring women’s perceptions of the factors that assisted them in breastfeeding for at least 6 months. Telephone interviews were conducted with 139 Swedish women who reported that they had breastfed for at least 6 months. Women were asked the question, *“Do you have any advice that you would like to give to healthcare professionals regarding breastfeeding support?”.* The data were analysed using content analysis.

**Results:**

The theme, “Professionals need to offer women sensitive, individualized breastfeeding support to promote a positive breastfeeding experience”, describes the women’s advice based on five categories: 1) providing evidence-based care, 2) preparing expectant parents during pregnancy, 3) creating a respectful and mutual dialogue, 4) offering individual solutions to breastfeeding problems, and 5) offering practical support.

**Conclusions:**

This study highlights the importance of professionals providing evidence-based breastfeeding support in a sensitive and individualized manner. This consideration is an important prerequisite to strengthening women’s self-confidence and assisting them in reaching their breastfeeding goals, which may enhance the positive nature of their breastfeeding experience.

## Background

Breastfeeding has short- and long-term health benefits for both women and their babies in low- and high-income countries. However, many women in high-income countries exclusively breastfeed for only a short period [1] and stop breastfeeding earlier than they would like [2, 3]. Exclusive breastfeeding for 6 months followed by continued breastfeeding with complementary food up to 2 years of age or beyond is recommended by the World Health Organization (WHO) [4]. The National Board of Health and Welfare in Sweden recommends exclusive breastfeeding for 6 months followed by continued breastfeeding up to 1 year of age or for as long as the woman and infant would like [5]. In Sweden, most women breastfeed exclusively for at least 1 week (78%), and approximately half of these women (51%) continue to breastfeed exclusively for at least 4 months. Few women breastfeed exclusively for 6 months (15%) or continue to breastfeed for 1 year (24%) [6].

Several socioeconomic factors have been shown to influence the length of the breastfeeding period in high-income countries; older mothers [7] and higher levels of education [1] and income are associated with breastfeeding for a longer period of time [1]. Hospital practices such as whether a newborn receives infant formula in the maternity ward [8], breastfeeding problems such as a perceived low milk supply, and the baby’s behaviour and health issues can have a negative influence on breastfeeding duration [2]. Psychosocial factors such as the intention to breastfeed, breastfeeding self-efficacy, and social support may also influence the duration of breastfeeding [9].

An important role of the Swedish healthcare system is to support breastfeeding [10, 11], and most families use the healthcare service, which is offered free of charge [11]. The concept of breastfeeding support is multidimensional and includes the aspects of supplying information, providing practical help, and addressing emotional and self-esteem issues. Informational support is given by providing information and advice. Breastfeeding women are supported emotionally when professionals care for them with a genuine, empathic approach. Support may also be provided by strengthening women’s self-confidence through encouragement and a positive attitude towards women’s own ideas [12].

Research from Sweden, Australia and Ireland has shown that women’s perceptions of what assisted them to breastfeed for at least 6 months are complex and multi-faceted. Women in all settings ranked informal face-to-face support and maternal self-determination as critical to continue breastfeeding for 6 months. Differences in ranking also appeared, with Irish women ranking informal online support higher than Australian and Swedish women. Swedish women ranked support from healthcare professionals such as midwives, nurses, and physicians first, and for Australian women, the most important factor was whether breastfeeding was going well [13]. Previous research carried out in Sweden and other high-income countries on women’s experiences of support from healthcare professionals has often focused on the first weeks after birth and has shown that women are often dissatisfied with the quality of breastfeeding support that they receive from healthcare professionals [10, 11, 14]. Synthesized findings have described breastfeeding support along two continuums: 1) from authentic presence to disconnected encounters and 2) from a facilitative to a reductionist approach. Authentic presence indicates that professionals adopt an empathetic and responsive approach rather than assigning blame and causing stress in the form of disconnected encounters. A facilitative style refers to providing accurate and sufficiently detailed information, while a reductionist approach refers to providing standard information or conflicting advice. From this perspective, women feel supported and helped with breastfeeding when professionals use a learner-centred approach with a trustful dialogue, provide realistic information, and offer practical help. In contrast, women can feel unsupported or pressured to breastfeed when professionals provide standard information or conflicting advice, when professionals do not have adequate time to spend with the women, or when practical support is provided in an intrusive and rough manner [14]. However, a Cochrane Review concluded that knowledge about what type of breastfeeding support women need from healthcare professionals to help them continue to breastfeed for an extended period is lacking [15]. Investigating women’s advice to healthcare professionals is important for the provision of optimal breastfeeding support.

### Aim

The aim of this study was to explore women’s advice to healthcare professionals regarding support for continuing to breastfeed for at least 6 months.

## Methods

### Study design

This investigation used an exploratory study design. This work is a follow-up of a larger study exploring women’s perceptions of the factors that assisted them in breastfeeding for at least 6 months [13].

### Setting

This study was conducted in Sweden, where antenatal care is provided by midwives during pregnancy and includes approximately 8–10 visits to the midwife. First-time parents are offered parental education. In general, women undergoing spontaneous vaginal birth receive postpartum care in a hospital for approximately 12 to 48 h. Newborns without complications are generally kept in skin-to-skin contact with their mothers for the first hour after birth. Parents often have their first contact with the child healthcare centre at approximately one to 2 weeks after birth, and they visit the child healthcare nurse approximately 12–13 times and the physician three times during the infant’s first year. If women experience any difficulties with breastfeeding, they can contact the breastfeeding outpatient clinics offered by some hospitals. Parents often have their first contact with a dental care provider when their babies are approximately 1 year of age. Dental care providers offer advice about oral health and tooth brushing.

### Study participants

The participants were women in Sweden who had breastfed for 6 months or longer. The women were still breastfeeding or had stopped within the previous 12 months.

### Data collection

Women were recruited through local newspapers and on social media platforms in the larger study [13]. Overall, 153 Australian women, 64 Irish women and 139 Swedish women were included in the larger study. This paper reports a follow-up study conducted with the 139 Swedish participants from the larger study. For this purposive sample, participants were recruited from four different social media platforms [16–19] between October 6, 2015, and January 21, 2016. The websites used are some of Sweden’s largest communities for families. These sites have information and discussion forums for expectant parents and parents who have newborns and older children. For example, the websites have forums for discussing pregnancy, childbirth, parenting, and feeding and nutrition (including infant formula feeding and breastfeeding). The women who were interested in participating followed a digital link to receive information on the study. To recruit participants by snowball sampling, an interviewer asked participants to share information about the study with other women who may meet the inclusion criteria [20]. Women who were interested in participating confirmed their interest to the first author (IB) by e-mail or telephone.

The first author collected data through audio-recorded telephone interviews in either Swedish or English. The interviews followed a semi-structured list of topics [20] and started with the question, “What has assisted you to continue breastfeeding to six months?”, followed by, “Could you rank the top three factors that you perceived were most important in assisting you to continue breastfeeding for at least six months?” [13]; a follow-up question used for this study was, *“Do you have any advice that you would like to give healthcare professionals regarding breastfeeding support?”* Probing questions included “Could you please explain in more detail?” The interview guide had previously been pilot-tested in five face-to-face interviews with women recruited through a child health centre. The pilot test resulted in minor language changes. These pilot interviews were included in the data analysis. The intention was to reach 100 women, and generally, up to 100 critical incidents are recommended according to the critical incident technique used in the larger interview study [20]. When 100 women were reached, the link was closed; however, 157 women had already expressed their interest by mail or text message.

In total, 162 women were interested in participating. Twenty-three women were excluded because they did not meet the inclusion criteria (they could not be reached after three attempts (*n* = 20), were not living in Sweden (*n* = 2) or had stopped breastfeeding more than 12 months ago (*n* = 1)). The final sample included 139 women who were interviewed at a time chosen by them. The interviews ranged from 10 to 56 min, with an average duration of 23 min. Of the interviewed women, 136 were interviewed in Swedish and three were interviewed in English.

### Analysis

Content analysis was used as described by Granheim et al. [21]. The interviews were transcribed verbatim by the first author, and the text was read several times to gain a sense of the whole. Statements related to the aim of the study (meaning units) were identified, and various colours were used to mark these meaning units in the text. The meaning units were condensed, abstracted, and assigned codes by the first author. Thereafter, the codes were managed in Excel. Codes from all interviews were compared and grouped into subcategories, initially by the first author and later together with two other members of the research team. The subcategories were compared and grouped into categories by the first author together with three others on the research team. In the last step, all five authors participated in the interpretation of the results. The codes were finally grouped into 20 subcategories and 5 categories. The categories described the content on a manifest level. Through reflective discussions with the research team, the underlying meaning of the categories was interpreted, and the following theme was developed: “*Professionals need to offer women sensitive, individualized breastfeeding support to promote a positive breastfeeding experience”*. This theme represented the interpreted latent content. During the analysis process, all context codes, subcategories, and categories and the theme were discussed until an understanding of the data was achieved. For an example of the analysis process, see Table 1. For details of the last step where the number of women who gave advice was counted, see Table 2 [22]. To present the sociodemographic characteristics of the women, we calculated descriptive statistics with SPSS (Table 3). For the women’s background factors in relation to the number of women who gave advice in each category, we calculated descriptive statistics with Excel (Table 4).

**Table 1:** Example of the analysis process

Considered meaning unit	Code	Subcategories	Categories	Theme
Be slightly more sensitive to the women’s needs	More sensitive to women’s needs	To be sensitive to women’s motivation and the family’s needs	Creating a respectful and mutual dialogue	Professionals need to offer women sensitive, individualized breastfeeding support to promote a positive breastfeeding experience
Listen to the woman, how she feels, and how breastfeeding is contributing to how she feels	Listen to how the woman feels			
Address different parents’ needs and be better at interpreting their needs	Assess different parents’ needs

**Table 2:** Overview of the categories and the theme and the number of women who gave advice^a^ in each category

Categories	Theme
Providing evidence-based care (*n* = 73)	Professionals need to offer women sensitive, individualized breastfeeding support to promote a positive breastfeeding experience
Offering individual solutions for breastfeeding problems (*n* = 55)	
Preparing expectant parents during pregnancy (*n* = 54)	
Creating a respectful and mutual dialogue (*n* = 49)	
Offering practical support (*n* = 34)	

(n refers to the number of women)

^a^Several women responded with multiple pieces of advice each

**Table 3:** Sociodemographic characteristics of the study participants

	Women *N* = 139 n (%)
Country of birth	
Sweden	118 (85)
Other	21 (15)
Level of education	
Compulsory school grades 1–9	5 (4)
High school	32 (23)
University	102 (73)
Civil status	
Married/cohabiting	133 (96)
Single or not living with a partner	6 (4)
^a^Age of the women (years)	33.5 (5.61) [20–51]
^a^ Number of children	1.8 (0.91) [1–6]
One	62 (45)
Two	51 (37)
Three or more	26 (19)
^a^Age in months of the youngest child	21.0 (11.2) [6–56]
Six to eighteen	69 (50)
Nineteen to thirty	48 (35)
Thirty-one to fifty-six	22 (16)
Feeding type for the youngest child at six months	
Exclusive breastfeeding	30 (22)
Human milk and formula	4 (3)
Human milk and solid food	95 (68)
Human milk and formula and solid food	10 (7)

^a^Mean (standard deviation)[range]

**Table 4:** Characteristics of participants and advice given in each category

Characteritics of the women		Categories									
		Providing evidence-based care *N* = 73		Offering individual solutions for breastfeeding problems *N* = 55		Preparing expectant parents during pregnancy *N* = 54		Creating a respectful and mutual dialogue *N* = 49		Offering practical support *N* = 34	
n		n	%	n	%	n	%	n	%	n	%
Country of birth											
Sweden	118	61	52	47	40	46	39	42	36	31	26
Other	21	12	57	8	38	8	38	7	33	3	14
Level of education											
Compulsory school grades 1–9	5	4	80	2	40	3	60	2	40	3	60
High school	32	19	59	15	47	15	47	7	22	8	25
University	102	50	49	38	37	36	35	40	39	23	23
Number of children											
One	62	41	66	26	42	32	52	24	39	20	32
Two	51	18	35	23	45	14	28	15	29	13	26
Three or more	26	14	54	6	23	8	31	10	39	1	4

(n refers to the number of women)

## Results

### Study participants

In total, 139 women gave advice to healthcare professionals regarding breastfeeding support. The categories and theme are summarised in Table 2. For the sociodemographic characteristics of the participants, see Table 3. For the women’s background factors in relation to the number of women who gave advice in each category, see Table 4.

### Sensitive individualized breastfeeding support

The women’s advice to healthcare professionals is described by the theme, “Professionals need to offer women sensitive, individualized breastfeeding support to promote a positive breastfeeding experience”. The theme emphasised the importance of healthcare professionals such as child healthcare nurses, physicians, midwives, and dental care staff providing evidence-based care in a respectful manner through a mutual dialogue based on individual needs.

### Providing evidence-based care

“Providing evidence-based care” was the category that encompassed the importance of up-to-date breastfeeding support. The women reported that professionals in the chain of healthcare service had various levels of knowledge regarding breastfeeding. The women were less satisfied with the support that they received after being discharged from a maternity facility after birth and at the child health centre than with the support that they received while hospitalized and from breastfeeding outpatient clinics. Women advised professionals to become more knowledgeable about breastfeeding and to improve their practical breastfeeding skills. For example:

*“It would be good if professionals knew more about breastfeeding. Often, you have to turn to volunteer organisations such as Amningshjälpen [a breastfeeding support group] because the child health centre does not know and public health services should know.” (code no. 41)*

*“I wish that they [the child health centre] were more well-read on breastfeeding because a lot of what I know about breastfeeding I have found out myself, and of course, the first thing that we do as new mums is turn to Google when we run into a problem, but there needs to be a deeper level of knowledge of breastfeeding problems.” (code no. 78)*

Dissatisfaction was experienced when breastfeeding recommendations were not consistent across the healthcare system or with the national and international guidelines. The women reported that they did not trust professionals who did not give evidence-based support. When they felt distrust, they did not listen to further recommendations or ask further questions. The following recommendations were given to professionals:

*“It is clearly stated on the Swedish National Food Agency’s website that there is no hurry before 6 months, that you can wait, that they [the child health centre] should read up on what is right, and that they stay updated.” (code no. 47)*

*“Refer to recommendations from the Swedish National Food Agency. The child health centre staff refer to their personal opinions when giving advice about breastfeeding. For example, she thought that my daughter should sleep by herself in her own bed and insisted that I should give my infant formula instead of breastfeeding at night.” (code no. 49)*

*“WHO promotes complementary breastfeeding for two years – they [healthcare professionals] need knowledge so that they can give the right information to mothers who want to continue to breastfeed after six months.” (code no. 95)*

The women advised the professionals to be positive and encouraging and to support mothers’ breastfeeding as long as they and their babies wanted to continue. Women often reported that they did not have sufficient support to be able to breastfeed exclusively, especially between four and 6 months. The women gave advice on how to support the continuance of breastfeeding in the complementary feeding period after 6 months. They did not want to feel pressured to stop breastfeeding at night, introduce solid food before 6 months or cease breastfeeding after a certain time, often after 6 months. When women were professionally advised to stop breastfeeding earlier than they wanted to, they became discouraged, sad, and disappointed because they had enjoyed breastfeeding. In this situation, the following advice was given by both primiparous and multiparous mothers:

*Do not talk about stopping breastfeeding in the night at four months, which gives a signal straight away that breastfeeding will stop soon.” (code no. 102)*

*“The child healthcare centre should not be discouraging us from breastfeeding. They [child healthcare nurses] started by telling me that he should start trying solids when he was four months, and then I came back when he was six months. How is breastfeeding going? Have you started cutting down? I think that you should because now the baby needs to eat more food.” (code no. 118)*

*“Health professionals should be encouraging. A doctor [at the child healthcare centre] gave me wrong information and told me that after six months, no nourishment was provided by my milk and I should stop." (code no. 65).*

*“My doctor [at the child healthcare centre] told me ‘you need to stop breastfeeding.’ Give people options before advice because a lot of people have to stop breastfeeding because the doctor tells them that they must stop, and then they are very sad.” (code no. 20)*

Some women questioned advice given by dental care staff about the cessation of night-time breastfeeding and breastfeeding cessation after 12 months. The woman indicated that they should be more informed about breastfeeding:

*“When he was one year old, my husband was at the dentist with him, and they recommended that he should not be breastfed at night. I asked what her education was that allowed her to give advice to all mothers to stop breastfeeding at night because such advice goes against WHO recommendations. It would be really great if dentists were more well read on the advice that they give.” (code no. 22)*

### Offering individual solutions for breastfeeding problems

This category featured statements about how professionals should support each woman individually. The problems mentioned were difficulties experienced with latching on, breastfeeding a tongue-tied baby, infant weight loss, painful breastfeeding, sore nipples, and mastitis. Women reported that they needed access to a calm, low-stress environment in the early months after birth, when experiencing breastfeeding as difficult is common. The women also reported that they needed continuous support because they did not always seek support when they had unresolved breastfeeding problems and experienced breastfeeding as difficult. One piece of advice emphasised the following practice:

*“Follow-up –the staff that you have met before should call and see how you are doing.” (code no. 56).*

Moreover, women advised professionals to be careful when giving general advice that may put pressure on women, such as “Just continue breastfeeding”, as many women needed support beyond the level of general advice to solve their breastfeeding problems. Professionals should encourage, confirm, and reassure women and trust them in their new role as breastfeeding mothers. The women advised professionals to have a trustful and supportive approach and to encourage women to continue breastfeeding and not to suggest using formula if not medically necessary. Two first-time mothers gave the following advice:

*“They should encourage us and give us more alternatives and not tell us straight away to start using formula.” (code no. 12)*

*“They [the child healthcare centre] only focus on the curve, and if the baby is not following the curve, they always say that you have to use formula, but maybe you can try other methods. You get so nervous as a mum thinking, ‘God, can I produce milk?’ You think that the baby will die if he does not put on weight.” (code no. 101)*.

A multiparous mother gave the following advice to child healthcare nurses at the child health centre:

*“Wait until the baby has been weighed a few times before saying to start with formula; give support.” (code no. 100)*

### Preparing expectant parents during pregnancy

The third category, “Preparing expectant parents during pregnancy”, included statements about how to prepare expectant parents for breastfeeding even during pregnancy. Women wanted additional breastfeeding information on various occasions during pregnancy. Information given at check-ups and during parental classes, both orally and written in leaflets or on websites, was recommended. Women advised healthcare professionals to include the partner, especially when the partner also wanted to be involved. A first-time mother gave the following advice to midwives:

*“Breastfeeding - more a part of the parenting course and also aimed at the fathers –how they can be part of breastfeeding and still have a good bond.” (code no. 36)*

Respondents said that professionals should inform expectant parents about available breastfeeding options to enable informed decisions about breastfeeding or formula feeding. The following advice was given to midwives:

*“When you go to the antenatal clinic, they ask you if you plan to breastfeed but nothing more on the subject. Convey information several times such that mothers and fathers understand the importance of breastfeeding, the convenience of not needing to warm bottles, and that breastfeeding becomes so much easier when you finally get it to work.” (code no. 101)*

*“More information – when parents do not have information, it is hard to know what we want” (code no. 114)*.

The women wanted professionals to inform them of the advantages of breastfeeding, such as its natural and convenient characteristics, the opportunity it offers to bond with the baby, and the economic and health benefits it provides for both the mother and the baby. A first-time mother gave the following advice:

*“The more I learnt about breastfeeding regarding nutrition and bonding, I understood that is important. I wish that they had managed to convey this information to us [expectant mothers and their partners] at the antenatal clinic. A lot of partners think that breastfeeding is not equal and that the dad is left out.” (code no. 40*).

Another mother gave the following advice:

*“Information on how important breastfeeding is – for the mother and baby’s health and bonding.” (code no. 88)*

Women also wanted professionals to give them realistic and practical information about how time-consuming the initiation of breastfeeding can be, approximately how often the baby needs to be breastfed, the baby’s behaviour, the physiology of breastfeeding, signs indicating that the mother has a sufficient milk supply, and how to increase milk production. The following comments are examples of statements about the information that women wanted during pregnancy:

*“Give expectant mothers a realistic picture of common problems” (code no. 8); “Tell mothers that a baby wanting to breastfeed often does not mean that not enough milk is available.” (code no. 2);* and *“Explain how the amount of milk increases with the frequency with which the baby feeds.” (code no. 105)*

### Creating a respectful and mutual dialogue

This category describes the importance of creating a sensitive and respectful dialogue with parents. Professionals were advised to have a respectful non-judgemental approach when giving breastfeeding support, especially with women who were experiencing an insufficient milk supply or who were not able to breastfeed exclusively. The following advice was given to nurses, physicians and midwives:

*“Speak positively to mothers.” (code no. 93)*

*“It would be nice if someone had said, ‘do not worry if it does not work right away; pump and feed and continue using this nipple shield and it will go well, and if it does not sort itself out and you must bottle-feed, then that will go well too,’ but there was guilt, I felt that a lot of blame was put on me.” (code no. 103)*

*“Do not shame mothers who cannot exclusively breastfeed.” (code no. 100).*

The women reported that they were dissatisfied when professionals gave too much advice or conflicting advice without listening to the family’s needs or focusing on nutrition. One piece of advice to professionals working at the maternity ward was as follows:

*“Listen to what parents think and feel about breastfeeding – I was given 30 unwanted pieces of advice.” (code no. 136).*

Healthcare professionals were advised to listen more and ask women questions about previous and current breastfeeding experiences, intentions and breastfeeding goals, expectations and what kind of breastfeeding support they wanted. Women acknowledged individual support based on their own preferences. Professionals were advised to spend the time needed and to use open-ended questions in the dialogue:

*“The physician asked if I had thought about breastfeeding versus bottle-feeding, and it was a good, open question, giving me the ability to say what I thought” (code no. 16)*; *“Ask, ‘How would you like me to help you?’” (code no. 135).*

### Offering practical support

The last category comprised statements about how professionals should give practical support: being calm and gentle and taking the time needed were considered important. After the birth of their babies, the women wanted to take the initiative to breastfeed. A mother described her experience in the following words:

*“Stand back, let the mother take the initiative with breastfeeding. Of course, they should help if they notice that the mother is wondering about something, but let it happen naturally for at least the first few hours." (code no. 26).*

Some women reported that they learned best when they put the baby to their breast by themselves. Healthcare professionals should avoid hands-on support and respect the bodies of both the woman and her baby when providing practical support*.* A first-time mother gave this advice to professionals:

*Work with a "hands-off“ approach – they [midwives] helped me position him [the baby] on the breast, but I had forgotten how to do it – then, I met a woman [midwife] who told me what to do, and it was so much better when I was able to do it myself.” (code no. 96).*

The results also showed that the presence of professionals during breastfeeding facilitated women’s ability to ask questions if needed. The preferred approach of healthcare professionals was described as follows by three first-time mothers:

*“Use a gentle, patient tone of voice when telling us how to breastfeed” (code no. 17)*; *“Sit down and observe how the baby is latching on” (code no. 111)*; and *“I would like for someone to show me and to take the time needed to do so and explain.” (code no. 104)*.

## Discussion

This interview study explored women’s advice to healthcare professionals on how to support women who are breastfeeding based on the participants’ own breastfeeding experiences. The women advised healthcare professionals to offer evidence-based breastfeeding support to enable women to reach their own breastfeeding goals. Healthcare professionals need to offer sensitive, individualized breastfeeding support through a respectful and mutual dialogue based on a family’s needs to encourage women to make informed decisions about continuing breastfeeding.

### Evidence-based individualized breastfeeding support

#### Preparing expectant parents during pregnancy

The women in the present study advised healthcare professionals to provide parents with evidence-based informal support for breastfeeding during pregnancy. The women felt supported when they and their partners received informal support about the advantages of breastfeeding, such as convenience, emotional closeness with the baby, and health benefits for both the mother and the baby. This support is consistent with the WHO guideline “Ten Steps to Successful Breastfeeding” [23]. The findings from this study supported the findings of Schmied et al. [14] describing women’s perceptions of breastfeeding support. They found that peers and professionals should use a facilitative style and provide accurate and sufficiently detailed information to help women feel in control of decisions regarding breastfeeding. The women in the present study said that professionals should notify expectant parents about available breastfeeding options to enable informed decisions about breastfeeding or formula feeding. They wanted realistic and practical informal support for initiating and carrying out breastfeeding. Previous qualitative studies showed that healthcare professionals gave expectant mothers unrealistic expectations of breastfeeding during antenatal care, which left them unprepared for early breastfeeding problems [24, 25]. Our study also shows that women wanted additional knowledge about the physiology of breastfeeding, indications of a sufficient milk supply, and how to increase milk production. The present study also found that women wanted healthcare professionals to involve their partners in the dialogues on breastfeeding during antenatal care so that they would not feel left out from the family relationship during breastfeeding. This finding is consistent with previous research showing the importance of involving the partner in breastfeeding for women’s satisfaction with support [24, 25]. However, a study from the UK found that partners often felt excluded from dialogues on breastfeeding during antenatal care and that they wanted to be more involved in consultations about breastfeeding to be better able to support their partners [26]. According to a Swedish study, women perceived support from their partners as crucial for strengthening their confidence in their ability to continue breastfeeding for 6 months. Importantly, partners should have a positive attitude towards breastfeeding and offer women encouragement and emotional support in their decision to continue breastfeeding [13].

#### Offering individual solutions for breastfeeding problems

Offering individual solutions for breastfeeding problems was a category that emerged during the analysis. The women wanted healthcare professionals to have a trustful and supportive approach and to provide relevant breastfeeding information to help mothers make informed decisions regarding breastfeeding versus formula feeding. Up to 60% of breastfeeding women stop earlier than they would like because they perceive that they have breastfeeding problems such as an insufficient milk supply or problems with their baby’s weight [2]. This study also shows that women need continuous support when they experience breastfeeding problems because they do not always seek support when they have unresolved breastfeeding issues. The results from our study support the findings of Schmied et al., who reported that women wanted proactive support [14]. A previous study from Sweden showed that 27% of breastfeeding women had breastfeeding problems in the first month after birth and that these problems were associated with early weaning [27]. This finding indicates that women in Sweden may not receive the support that they need to continue breastfeeding when they experience breastfeeding problems.

#### Offering practical support

One finding from this study was that healthcare professionals should provide women with practical breastfeeding skills. One woman in the present study reported that her learning experience was improved when healthcare professionals used hands-off practical breastfeeding support techniques, which may strengthen women’s self-confidence about breastfeeding. In a meta-synthesis, Schmied et al. found that women wanted breastfeeding support that helped them learn for themselves [14]. One systematic review describes professionals showing trust in both the mother’s and baby’s ability to breastfeed when providing hands-off breastfeeding support [28]. In contrast, a study from Sweden indicated that hands-on breastfeeding support from healthcare professionals may undermine women’s self-confidence about breastfeeding and lead to feelings of existential insecurity and fear [29]. A previous study from Sweden showed that a hands-on approach is commonly used in the maternity ward [30]. Midwives in a maternity ward often stated that they wanted to use hands-off techniques but were too rushed to use this approach when giving practical support [28].

#### Creating a respectful and mutual dialogue

Furthermore, the analysis shows that healthcare professionals’ communication skills are important. Similar findings have been reported by Schmied et al., who found that healthcare professionals need good communication skills when offering individualized breastfeeding support for women to be satisfied with the support [14]. Burns et al. showed that healthcare professionals’ communication skills were important for strengthening women’s autonomy [31]. Having autonomy in the context of breastfeeding has been described as important for women’s experiences of control over their own bodies and lives [32]. The women in the present study, who had all breastfed for at least 6 months, preferred to have healthcare professionals use open-ended questions and listen to their experiences, intentions and goals in relation to breastfeeding. Other studies found that the use of open-ended questions gives women the opportunity to lead dialogues on breastfeeding without feeling pressured about breastfeeding [24, 28]. In our study, the subjects perceived healthcare professionals as having a judgemental approach towards women who were experiencing an insufficient milk supply or who were not able to exclusively breastfeed. When women start to use infant formula, they may feel negatively judged by healthcare professionals [33], and such healthcare professionals must be aware of how they may impose guilt. In fact, Schmied et al. found that women feel blamed when they cannot breastfeed [14]. We argue that healthcare professionals must consider the importance of developing trustful interactions with breastfeeding women by applying a sensitive, non-judgemental approach.

#### Providing evidence-based care

The women in the present study reported that healthcare professionals need to be knowledgeable about breastfeeding. According to our study, women perceived that healthcare professionals who care for women after they are discharged from a maternity facility and during the first months after childbirth need additional knowledge and practical skills to support women in solving their own breastfeeding problems. The WHO guideline “Ten Steps to Successful Breastfeeding” indicates that healthcare professionals should have sufficient knowledge, competence and skills to support breastfeeding [23]. The growth charts currently used in Sweden are based on babies fed infant formula. Healthcare professionals may need additional knowledge about the WHO’s growth charts for healthy breastfed babies [34] to provide evidence-based care to breastfed babies. This weakness needs to be considered because a lack of professional support is related to women feeling pressured to breastfeed or women breastfeeding for only a short duration [24, 35].

The recommendation from the Swedish women in this study to professionals was to provide up-to-date support based on the national and international guidelines [4, 5]. Such knowledge includes information on alternatives to discontinuing night-time breastfeeding and to the introduction of solid foods before 6 months. The women in our study are well informed about breastfeeding and the national and international guidelines and may seek information, for example, on the internet. The WHO and Sweden recommend exclusive breastfeeding for 6 months [4, 5]. However, the National Food Agency in Sweden informs parents that once the baby reaches 4 months of age, they can let the baby taste small samples of solid food if the baby seems interested [36]. Additionally, The National Board of Health and Welfare in Sweden also recommends that healthcare professionals should advise parents that they should let the baby sleep in its own bed during the first 3 months after birth to reduce the risk of sudden infant death (SIDS) [37]. These sources of information may seem conflicting when healthcare professionals provide breastfeeding advice. However, a study from Sweden found that nurses at a child health centre often have a negative attitude towards exclusive breastfeeding for six months. The nurses conveyed that parents should be advised to introduce solid food when the baby is four months old because if they wait, the baby will not be willing to eat solid food. The professionals also perceived night-time breastfeeding as tiring for women and that women should prolong breastfeeding intervals during the night [38]. Appropriate informal support is important for women to be satisfied with the support that they receive during the childbearing period [39]. Dykes reports that nurses, physicians, and midwives must develop a socio-cultural understanding of breastfeeding [40]. We agree with Tomori et al., who suggest that healthcare professionals have a considerable influence on a woman’s decision to continue breastfeeding due to their position of authority [41].

According to the present study, encouragement and a positive approach by healthcare professionals were important factors in women’s continuing to breastfeed. Professionals were advised to strengthen women’s self-confidence in breastfeeding such that they could make their own choices and reach their breastfeeding goals. The WHO recommends continued breastfeeding with complementary food up to 2 years of age or beyond [4]. Sweden recommends continued breastfeeding up to 1 year of age or for as long as the mother and infant would like [5]. In our study, we found that women did not want to feel pressured to cease breastfeeding after a certain time, which is often 6 months in practice. Women also questioned breastfeeding advice given by dental care staff about the cessation of night-time breastfeeding or breastfeeding cessation after 12 months. These findings have important implications for professionals, as this study shows that the women in the present study who needed to wean earlier than they would have liked became sad and disappointed. Professionals must respect and support women’s own breastfeeding decisions. This study found that the healthcare system can be improved by offering professional individual support. Importantly, women must reach their own breastfeeding goals to have a positive breastfeeding experience because breastfeeding has important health benefits for both women and their babies [1].

### Methodological considerations

A limitation of the study was that the interviews were short in duration, which may have limited the depth of the analysis. Another potential limitation of the study is related to three drawbacks of telephone interviews: they may create distance between the interviewer and the interviewee, a risk of miscommunication exists, and changes in body language are lost [42]. A suitable alternative may be self-administered questionnaires. The research team’s prior understanding of the topic may have influenced the research process as well as the results. The research team in this study contains multiple professionals, including four midwives and one physician, and the analysis was discussed by the team of researchers together with clinical nurses, child healthcare nurses and midwives; thus, attention was focused on the research team’s prior understanding, which may have influenced the research process as well as the results [21]. Discussions regarding the theme, categories, and subcategories continued until an agreement was reached, and quotes gave voices to the participants in this study [21]. Most (73.4%) of the women in this study had a university education. In Sweden, 44% of women who gave birth to a child in 2017 had a university education according to the Swedish Pregnancy Register. Additionally, fewer women in our study were born outside of Sweden (15.1%) than the number reported in the Swedish Pregnancy Register of 2017 (36%) [43]. The Swedish women included in the study were self-selected from social media platforms and had all continued to breastfeed for at least 6 months. The findings may represent breastfeeding women who have especially high motivation, breastfeeding self-efficacy and confidence in breastfeeding [44]; a broader range of participants may provide different views.

One strength of this study was that we recruited study participants from four different social media platforms to include women with various breastfeeding experiences and explore the research question from different aspects [21]. Additionally, we chose to include women who were still breastfeeding or who had stopped within the previous 12 months to reduce maternal recall bias, since a period of 3 years or less has been recommended when focusing on baby feeding practices [45].

## Conclusion

This study highlights that breastfeeding is often considered a personal choice of the mother and that the responsibility for breastfeeding often rests heavily on her shoulders. The message that breastfeeding has health benefits is not accompanied by evidence-based and responsive support to facilitate breastfeeding. In fact, interviews with mothers revealed that breastfeeding is difficult for women in a society that does not completely embrace a positive attitude towards breastfeeding. Breastfeeding advice is often conflicting and impossible to follow.

The study shows the importance of professionals providing evidence-based breastfeeding support in a sensitive and individualized manner. Women feel confused when they receive conflicting messages from the national and international guidelines and healthcare professionals about breastfeeding. This information is an important prerequisite to strengthening women’s self-confidence and assisting them in reaching their breastfeeding goals, which may enhance the positive nature of the breastfeeding experience.

## Data Availability

The Swedish transcripts and SPSS data file are not available in English and are not publicly available for ethical reasons but are available from the corresponding author upon reasonable request.

## Abbreviations

SIDS: Sudden infant death
WHO: World Health Organization
